# Cognitive Stress Regulation in Schizophrenia Patients and Healthy Individuals: Brain and Behavior

**DOI:** 10.3390/jcm12072749

**Published:** 2023-04-06

**Authors:** Lydia Kogler, Christina Regenbogen, Veronika I. Müller, Nils Kohn, Frank Schneider, Ruben C. Gur, Birgit Derntl

**Affiliations:** 1 Department of Psychiatry and Psychotherapy, Tübingen Centre for Mental Health (TüCMH), Medical Faculty, University of Tübingen, Calwerstrasse 14, 72076 Tübingen, Germany; 2 Department of Psychiatry, Psychotherapy and Psychosomatics, RWTH Aachen University, Pauwelsstraße 30, 52074 Aachen, Germany; 3 Institute of Neuroscience und Medicine, INM-7, Research Centre Jülich, 52425 Jülich, Germany; 4 Institute of Systems Neuroscience, Medical Faculty, Heinrich Heine University Düsseldorf, Moorenstraße 5, 40225 Düsseldorf, Germany; 5 Donders Institute for Brain, Cognition and Behaviour, Radboud University Medical Centre, Postbus 9101, 6500 HB Nijmegen, The Netherlands; 6 Medical Faculty, Heinrich Heine University Düsseldorf, Moorenstraße 5, 40225 Düsseldorf, Germany; 7 Neuropsychiatry Division, Department of Psychiatry, University of Pennsylvania Perelman School of Medicine, Philadelphia, PA 19104, USA; 8 International Max Planck Research School for the Mechanisms of Mental Function and Dysfunction (IMPRS-MMFD), Otfried-Müller-Str. 27, 72076 Tübingen, Germany; 9 LEAD Graduate School and Network, University of Tübingen, Walter-Simon-Straße 12, 72074 Tübingen, Germany

**Keywords:** emotion regulation, ventral ACC, IFG/VLPF, insula, parietal cortex, amygdala, hippocampus, stress, schizophrenia

## Abstract

Stress is an important factor in the development, triggering, and maintenance of psychotic symptoms. Still, little is known about the neural correlates of cognitively regulating stressful events in schizophrenia. The current study aimed at investigating the cognitive down-regulation of negative, stressful reactions during a neuroimaging psychosocial stress paradigm (non-regulated stress versus cognitively regulated stress). In a randomized, repeated-measures within-subject design, we assessed subjective reactions and neural activation in schizophrenia patients (SZP) and matched healthy controls in a neuroimaging psychosocial stress paradigm. In general, SZP exhibited an increased anticipation of stress compared to controls (*p* = 0.020). During non-regulated stress, SZP showed increased negative affect (*p* = 0.033) and stronger activation of the left parietal operculum/posterior insula (*p* < 0.001) and right inferior frontal gyrus/anterior insula (*p* = 0.005) than controls. Contrarily, stress regulation compared to non-regulated stress led to increased subjective reactions in controls (*p* = 0.003) but less deactivation in SZP in the ventral anterior cingulate cortex (*p* = 0.027). Our data demonstrate stronger reactions to and anticipation of stress in patients and difficulties with cognitive stress regulation in both groups. Considering the strong association between mental health and stress, the investigation of cognitive regulation in individuals vulnerable to stress, including SZP, has crucial implications for improving stress intervention trainings.

## 1. Introduction

Stressful situations elicit modulatory behavioral and psychophysiological reactions [1] and are thought to play a role in the development and maintenance of schizophrenia symptoms [2]. Certain biological and genetic vulnerabilities, in addition to environmental factors, make some individuals more susceptible to the triggering effects of stress, which can in turn contribute to the development of psychosis and positive symptoms of schizophrenia [2,3,4]. Schizophrenia patients (SZP) are thought to have an increased sensitivity and perception of stress, consequently affecting social-cognitive functioning [2,3]. Additionally, on a neural level, alterations in stress reaction in SZP have been reported in regions associated with affect and stress regulation: in a psychosocial stress task, reduced activation of the anterior cingulate cortex (ACC) and significant associations between amygdala and hippocampus activation, performance and subjective stress reactions were observed [5]. Similarly, in individuals who are prone to negative symptoms of schizophrenia, deactivations of the ACC and the amygdala were reported during psychosocial stress [6]. 

The experience of stress can be altered by cognitive strategies that modulate the negative subjective experience of emotions [7]. The ability to cognitively regulate emotions is of high adaptive value for mental health [8]. However, stress also impacts these regulation abilities [9,10]. In schizophrenia, a significant impairment in emotion regulation has been reported, but only a few studies have investigated the neural correlates of emotion regulation in schizophrenia [11,12,13]. During the downregulation of negative affect in response to emotional pictures, patients compared to controls showed less activation in the inferior frontal gyrus (IFG)/ventrolateral prefrontal cortex (VLPFC) and the insula [11,13], but an increased activation in the IFG/VLPFC and ACC was observed when upregulating or maintaining negative affect [11].

Summarizing previous literature, SZP show (1) altered stress reactions and (2) disturbed patterns in regulating negative experiences, which are present on a subjective level and in neural regions associated with affect and stress processing. It seems that SZP have dysfunctions in cognitively regulating negative experiences, but the knowledge on cognitive regulation of psychosocial stress in schizophrenia is sparse. Within the current study, we want to examine the regulation of psychosocial stress in more detail in schizophrenia patients. Extending the knowledge base on stress regulation has crucial implications for schizophrenia patients, as coping with stress affects further social-cognitive domains, and the improvement of treatments for individuals vulnerable to stress is necessary. 

Within the current study, we instructed healthy individuals and SZP to either (a) cognitively regulate or (b) maintain their negative sensations in stressful, psychosocial situations [7] to examine cognitive stress regulation. Based on previous reports [5,10,11,13], we hypothesized that the application of regulation techniques in stressful situations would lead to increased subjective stress in all participants. We further expected that regulating psychosocial stress would affect neural activation in SZP, particularly in those regions that have been reported to show aberrant functions during stress processing or emotion regulation, such as the IFG, insula, ACC, amygdala, and hippocampus.

## 2. Materials and Methods

### 2.1. Sample

Initially, 23 SZP participated in the experiment. Patients fulfilled ICD-10 diagnoses (given by the corresponding psychiatrist) and additionally met DSM-criteria for schizophrenia [14]. One female patient discontinued the experiment due to the stressful experience, and two patients were excluded due to their schizoaffective disorder. Hence, the final sample consisted of 20 schizophrenia patients (7 women) and 20 age- and gender-matched healthy controls (HC, 7 women); the control sample partly overlaps with the sample of [10]. All participants were right-handed [15]. HC reported no history of neurological or mental disorders [14]. Written informed consent was obtained from each participant prior to the experimental procedure. Participants were financially reimbursed after completion (€10/h). The authors assert that all procedures contributing to this work comply with the ethical standards of the relevant national and institutional committees on human experimentation and with the Helsinki Declaration of 1975, as revised in 2008. All procedures involving human subjects were approved by the ethics committee of the Medical Faculty of the RWTH Aachen University (approved IRB number EK05/12). 

### 2.2. Neurocognitive Abilities and Questionnaires

In order to control potential group differences based on neurocognitive abilities, participants were administered neuropsychological tests tapping verbal intelligence (WST) [16], executive functions (TMT) [17], and working memory (digit span; WIE) [18]. Moreover, questionnaires assessing stress coping (CISS) [19], emotion regulation strategies (ERQ) [20], and depression (BDI-2) [21] were completed. In SZP, positive and negative symptoms [22,23], clinical parameters, and medication were assessed.

### 2.3. fMRI Stress Task

**Figure 1:**
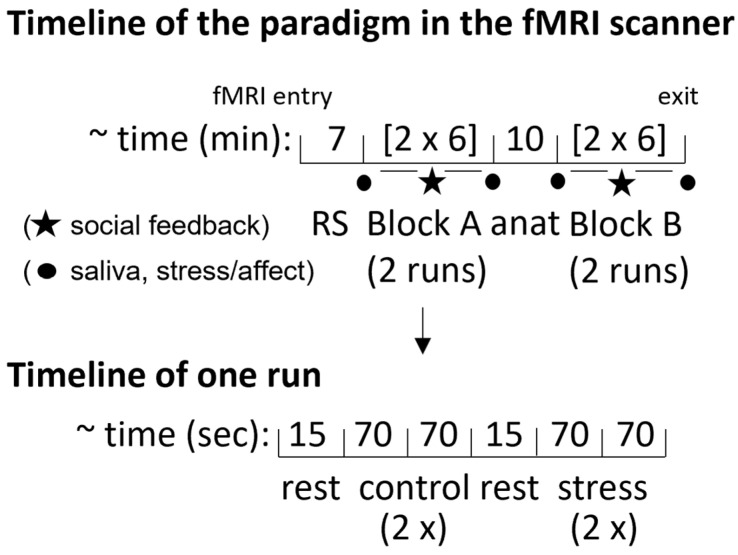
Timeline of the paradigm within the fMRI scanner and timeline for one run. Block A and Block B were either the non-regulation or the regulation block. The non-regulation and regulation blocks were presented in a randomized order across participants. A resting-state scan (RS) and an anatomical scan (anat) were assessed before Block A and Block B to adapt subjective arousal levels before each block.

We applied the “Montreal Imaging Stress Test” (MIST) [24,25], which has frequently been used in neuroimaging studies. Please also see [10] for a detailed task description. The experiment consisted of three conditions (rest, control, and stress), each performed under two different strategies (non-regulation and regulation). In total, two blocks (non-regulation, regulation block) with two runs per block and two rest (15 s), two control (70 s), and two stress (70 s) conditions per run were conducted (Figure 1). Between conditions, a jittered fixation cross (6–8 s) was presented. During rest, participants watched the screen (no arithmetic tasks were shown), during the control condition they performed mental arithmetic tasks, and during the stress condition an additional (adaptive) time limit to the arithmetic tasks (based on the participant’s performance) was presented (cf. [24]). Between runs the investigator gave feedback on the performance, told participants about the necessity of the specific performance level and asked them to improve their performance. The two blocks (non-regulation and regulation) were presented in a randomized order: for the non-regulation block, participants should simply perform the task and were told that their performance level is important for subsequent data analysis. For the regulation block, participants were instructed to regulate their negative sensations in a top-down attentional manner. In a pre-experimental training session, participants received training on the mental arithmetic tasks and were familiarized with the regulation instruction (cf. [10]). The investigator explained that stressful situations can be perceived in different ways. Participants were asked to think about the way they felt during their training on mental arithmetic tasks. They were trained to modify their negative sensations, to place less emphasis on the time limit and the negative performance feedback, and to focus instead on their intrinsic motivation to improve their performance since this was still important for subsequent data analysis. Thereby, participants were instructed to modify their negative sensations about the external factors of the paradigm in such a way that these factors were no longer experienced as stressful. Participants were debriefed after the regulation training, and all participants confirmed that they understood and could apply the regulation instruction when told to do so. Participants provided self-ratings on subjective stress and affect [26] before and after each block. After completion of the paradigm, subjects indicated whether they were able to regulate their stress reaction. Skin conductance response (SCR) during the task and salivary cortisol samples before and after each block were assessed to compare physiological activity due to stress regulation between groups. Analyses and results for cortisol and SCR are presented in the Supplementary Materials. 

### 2.4. Statistical Analysis of Behavioral and Psychophysiological Data

Statistical analysis was conducted using SPSS 24.0 (IBM Corp., Armonk, NY, USA). To test the effect of stress vs. stress regulation between groups, behavioral performance in the MIST (percent of correct trials in the stress condition), subjective stress, and negative affect were analyzed using repeated-measures analysis of variances (rmANOVAs) with the following factors: For performance, a 2 × 2 rmANOVA with the within-subject factor “regulation” (non-regulation/regulation) and the between-subjects factor “group” was performed. For subjective stress and negative affect, separate 2 × 2 × 2 rmANOVAs with the within-subject factors “regulation” and “time” (pre-stress/post-stress), the between-subject factor “group”, and the covariate “cognitive flexibility” (TMT-B-values) were performed. One patient was excluded from the analysis as the TMT-B-value (181 s) exceeded two standard deviations from the mean. For two healthy individuals, the subjective data was not logged due to software problems. Analyses for cortisol and SCR can be found in the Supplementary Materials. The level of significance was set at *p* < 0.05 for all tests. Degrees of freedom were adjusted using Greenhouse-Geisser-correction, if necessary. Significant effects were examined with planned simple contrasts and post-hoc *t*-tests. Partial eta-squared (ηp^2^) is reported for significant results in rmANOVAs.

### 2.5. Imaging Data Acquisition

The fMRI data were acquired using a 3T Siemens Trio Scanner (Siemens Medical Systems, Erlangen, Germany) located at the Department of Psychiatry, Psychotherapy, and Psychosomatics, RWTH Aachen. A standard head coil and foam padding were used to reduce head motion. T2*-weighted echo-planar image (EPI) sequences (34 slices, TR = 2000 ms, TE = 28 ms, gap 10%, flip-angle = 77°, voxel: 3.6 × 3.6 × 3.3 mm) were acquired in an axial plane. Prior to the paradigm, 210 images taken during a resting state were assessed. Additionally, T1 anatomical images were acquired (MPRAGE, TR = 1900; TE = 2.52; TI = 900 ms; flip-angle = 9°; 256 matrix; FoV = 250 mm; 176 slices per slab; voxel: 1 × 1 × 1 mm, 9 min 50 s). 

### 2.6. fMRI Preprocessing and Analysis

The fMRI data were analyzed using SPM12 (Wellcome Department of Imaging Neuroscience, London, UK) implemented in Matlab (Mathworks Inc., Sherborn, MA, USA). Five dummy scans at the beginning of the experiment were discarded. Pre-processing comprised reorientation of the functional images, slice-time correction, head motion correction using realignment, and coregistration to the mean anatomical image. The data was subsequently normalized to the Montreal Neurological Institute (MNI) space using a segmentation algorithm [27]. Images were resampled in the MNI space at a 2 × 2 × 2 mm voxel size and spatially smoothed using a 6 mm full-width-at-half-maximum Gaussian kernel. Functional data were analyzed using a general linear model. Each of the six conditions (rest, control, and stress for both the non-regulation and the regulation block) was separately modeled by a boxcar reference vector convolved with a canonical hemodynamic response function and its first-order temporal derivate. Scan movement parameters as estimated during spatial realignment were included as covariates of no interest. A high-pass filter (512 Hz) was applied. Simple main effects for each of the conditions were computed for every participant and then fed into a second-level group analysis using a mixed-effects model (factor: condition, subject). As group differences in performance appeared, the percent of correct trials in the paradigm was added as a covariate. We modeled 6 experimental conditions (non-regulation: rest, control, stress; regulation: rest, control, stress) for 2 groups (SZP and HC). All effects were at a threshold of *p* < 0.05 at the cluster level, familywise error-corrected for multiple comparisons (pFWE < 0.05), with a cluster-forming threshold of *p* < 0.001, uncorrected. An extent threshold of k = 99 contiguous voxels corresponds to a corrected threshold of *p* < 0.05. All results are reported at this cluster-level corrected threshold. For whole-brain effects, the SPM AnatomyToolbox (v2.2b) [28] was used for anatomical localization. 

### 2.7. Region-of-Interest Analysis

Based on previous results [5,10,11,13], we defined a priori hypotheses and performed region-of-interest (ROI) analyses for the following regions: right IFG/anterior insula (aI), bilateral ventral ACC, hippocampus, and amygdala. Anatomical ROIs for the amygdala, hippocampus, and ACC were created with the SPM AnatomyToolbox (v2.2b) [28]. The cluster of the IFG/aI was extracted from [29]. The mean values of the specific clusters within these ROIs were extracted from the paradigm data for each block (non-regulation/regulation) and each condition (rest/control/stress) and fed into rmANOVAs (factors “laterality”, “regulation”, “condition”, and “group”). ROI and additional exploratory regression analyses (medication, symptom severity, subjective stress; see Supplementary Materials) were performed using SPSS 24.

## 3. Results

### 3.1. Sample Description

Sample characteristics are listed in Table 1. Group differences were seen in working memory, processing speed, and cognitive flexibility, with better performance in HC compared to SZP. Due to these differences, we included cognitive flexibility as a covariate in subjective analyses (as working memory and processing speed are also subsumed under this function) [30]. Patients also had higher depression scores compared to HC (t28.614 = 4.694, *p* < 0.001).

### 3.2. Behavioral Results

The rmANOVA showed a significant main effect of “group” (F1; 38 = 9.863, *p* = 0.003, ηp^2^ = 0.206), with HC performing better (more correct trials) than SZP. Neither the main effect “regulation” nor the interaction was significant (all *ps* > 0.172). 

### 3.3. Subjective Ratings 

Inquiries following the paradigm indicated that 27 participants (16 SZP) were able to modify the negative experience during the task, whereas 9 were not (3 SZP) (four individuals did not complete the item, thus no data is available). 

#### 3.3.1. Subjective Stress

**Figure 2:**
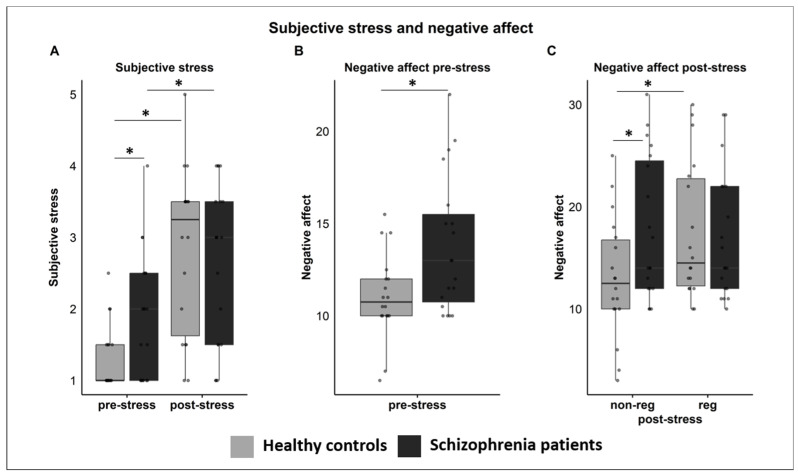
Illustration of significant interactions as revealed by the ANOVAs for subjective ratings. (**A**) Subjective stress, interaction “time-by-group”: SZP compared to HC had higher subjective stress before the stress paradigm. Subjective stress increased in both groups from before to after the stress paradigm. (**B**,**C**) are disentangling the 3-way interaction “regulation-by-time-by-group” for negative affect. (**B**) Pre-stress, group main effect: SZP compared to HC had higher negative affect before the stress paradigm. (**C**) Post-stress, interaction “regulation-by-group”: SZP had higher negative affect than HC after the non-regulation block. HC reported higher negative affect after the regulation compared to the non-regulation block. No significant group difference appeared after the regulation block. non-reg = non-regulation block; reg = regulation block. Significant differences are marked with *.

The rmANOVA revealed a significant interaction effect for “time-by-group” (F1; 34 = 7.325, *p* = 0.011, ηp^2^ = 0.177), with higher levels of stress for SZP than HC before stress (*p* = 0.020), whereas no such difference appeared after stress (*p* = 0.643). Within both groups, subjective stress increased from before to after stress (HC: *p* < 0.001; SZP: *p* = 0.003) (Figure 2A). No other main effect or interaction was significant (all *ps* > 0.08). 

#### 3.3.2. Negative Affect

The rmANOVAs revealed a significant 3-way interaction of “regulation-by-time-by-group” (F1; 34 = 7.723, *p* = 0.009, ηp^2^ = 0.185). No other main effect or interaction was significant (all *ps* > 0.06). To further disentangle the 3-way interaction, separate rmANOVAs were performed before and after stress (factors: “regulation” and “group”). Before stress, results revealed a significant main effect for “group” (F1; 34 = 8.345, *p* = 0.007, ηp^2^ = 0.197), with higher negative affect for SZP than HC (Figure 2B). After stress, no significant main effect occurred (all *ps* > 0.419). However, a significant interaction emerged (F1; 34 = 12.096, *p* = 0.001, ηp^2^ = 0.262): After the non-regulation block, SZP reported significantly higher negative affect than HC (*p* = 0.033), whereas no group difference appeared after the regulation block (*p* = 0.835). Furthermore, HC reported higher negative affect after the regulation than the non-regulation block (*p* = 0.003), whereas no such difference appeared within SZP (*p* = 0.301) (Figure 2C).

Ratings for subjective stress and negative affect are listed in Table 2 and depicted in Supplementary Figure S1 (see Supplementary Materials).

### 3.4. Whole-brain fMRI Data

**Figure 3:**
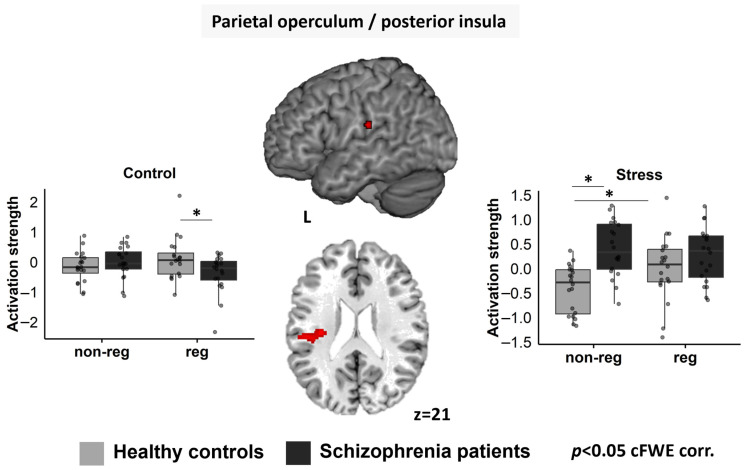
The cluster in the pOP/pI (red) showed a significant interaction “regulation-by-condition-by-group” on whole-brain level: HC showed stronger activation than SZP during the control condition in the regulation block (**left**). SZP showed higher activation than HC during the stress condition in the non-regulation block (**right**). HC showed higher activation during stress in the regulation block compared to the non-regulation block (**right**). non-reg = non-regulation block; reg = regulation block; L = left. Significant differences are marked with *.

Details for the whole-brain analyses for each group separately for activation in the control and stress conditions within each block (non-regulation, regulation) can be found in the Supplementary Materials (Table S1). An F-test for the interaction regulation-by-condition-by-group revealed a significant cluster in the left parietal operculum (pOP) extending into the posterior insula (pI) (Figure 3). The mean values of this cluster were extracted, and post-hoc analyses were run with SPSS to characterize the cluster in more detail: 

#### Left pOP/pI

To split up the 3-way interaction observed at whole-brain level (Figure 3), we conducted rmANOVAs (factors “regulation” and “group”) for each condition (rest/control/stress) separately: 

For the rest condition, a significant main effect was seen for “group” (F1; 38 = 17.027, *p* < 0.001, ηp^2^ = 0.309), with higher activation in SZP than HC. 

For the control condition, there were no significant main effects (all *ps* > 0.188), but a significant interaction was seen (F1; 38 = 5.108, *p* = 0.030, ηp^2^ = 0.118). Post-hoc *t*-tests showed significantly higher activation in SZP during the regulation block (*p* = 0.027), while no group difference emerged for the non-regulation block (*p* = 0.330) (Figure 3). 

For the stress condition, a significant main effect was seen for “group” (F1; 38 = 20.535, *p* < 0.001, ηp^2^ = 0.351), with higher activation in SZP than HC. Furthermore, a significant interaction appeared (F1; 38 = 4.092, *p* = 0.050, ηp^2^ = 0.097). During the stress condition in the non-regulation block, SZP showed significantly higher activation than HC (*p* < 0.001), whereas no group difference was seen during the regulation block (*p* = 0.176). Furthermore, HC showed significantly higher activation in the regulation block than the non-regulation block (*p* = 0.040), but no difference was observed within SZP (*p* = 0.511) (Figure 3).

### 3.5. Region-of-Interest Analyses

For the sake of conciseness in the current manuscript, we only report interactions, including regulation and group, within the main manuscript. Therefore, results for the amygdala and hippocampus are described in the Supplementary Materials. 

#### 3.5.1. ACC

**Figure 4:**
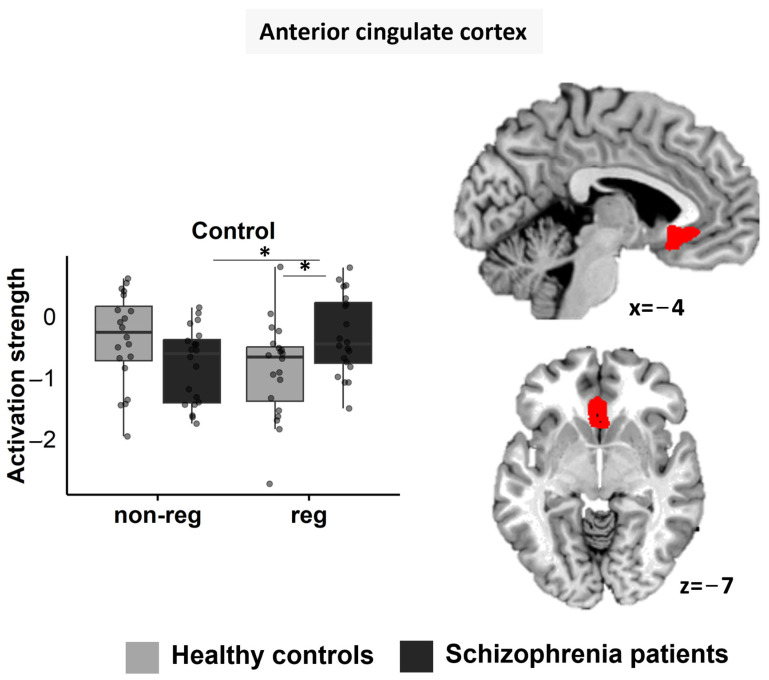
Region-of-interest analysis of the ventral ACC (red) showed a “regulation-by-condition-by-group” interaction. While no group differences emerged for the stress condition, SZP showed less deactivation compared to HC in the control condition in the regulation block. Additionally, in SZP, the ventral ACC was less deactivated in the control condition in the regulation block compared to the control condition in the non-regulation block. non-reg = non-regulation block; reg = regulation block. Significant differences are marked with *.

Using rmANOVA, significant main effects emerged for “regulation” (F1; 38 = 5.156, *p* = 0.029, ηp^2^ = 0.119) with less deactivation in the regulation compared to the non-regulation block, for “condition” (F2; 76 = 6.606, *p* = 0.002, ηp^2^ = 0.148) with less deactivation in the stress (*p* = 0.012) and the rest (*p* < 0.001) compared to the control condition, as well as for “group” (F1; 38 = 7.234, *p* = 0.011, ηp^2^ = 0.160) with less deactivation in SZP than in HC. Furthermore, a significant interaction was seen for “regulation-by-condition-by-group” (F2; 76 = 3.158, *p* = 0.048, ηp^2^ = 0.077) (Figure 4). To split up the 3-way interaction, we conducted three separate rmANOVAs for each condition (rest/control/stress) (factors “regulation” and “group”). 

For the rest condition, a significant main effect appeared for “group” (F1; 38 = 6.289, *p* = 0.017, ηp^2^ = 0.142), with deactivation in HC while SZP showed activation. No other effect was significant (all *ps* > 0.222). 

For the control condition, no significant main effect appeared (all *ps* > 0.679), but the interaction “regulation-by-group” was significant (F1; 38 = 8.258, *p* = 0.007, ηp^2^ = 0.179) (Figure 4). Post-hoc *t*-tests showed less deactivation of the ACC in SZP compared to HC in the regulation block (*p* = 0.021) and less deactivation of the ACC within SZP in the regulation block compared to the non-regulation block (*p* = 0.027). There was no significant difference between groups in the non-regulation block (*p* = 0.055), and for HC, the non-regulation and regulation blocks did not differ (*p* = 0.088). 

For the stress condition, a significant main effect for “regulation” emerged (F1; 38 = 8.187, *p* = 0.007, ηp^2^ = 0.177), with less deactivation in the regulation block compared to the non-regulation block. No other effect was significant (all *ps* > 0.164). 

#### 3.5.2. Right IFG/aI

**Figure 5:**
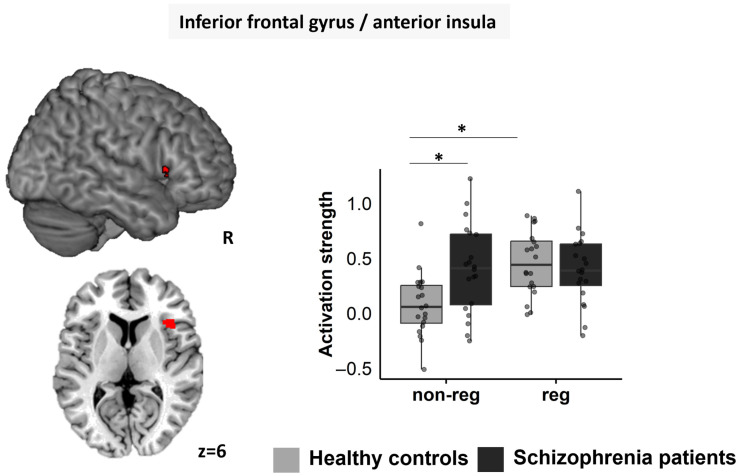
Region-of-interest-analysis of the right IFG/aI (red) showed a “regulation-by-group” interaction. SZP showed higher activation than HC within the non-regulation block. For HC, higher activation was seen in the regulation block compared to the non-regulation block. non-reg = non-regulation block; reg = regulation block; R = right. Significant differences are marked with *.

The rmANOVA revealed significant main effects for “regulation” (F1; 76 = 7.598, *p* = 0.009, ηp^2^ = 0.167) with higher activation during the regulation than the non-regulation block, and “condition” (F1; 76 = 60.360, *p* < 0.001, ηp^2^ = 0.614) with higher activation during stress than control (*p* = 0.001) and rest (*p* < 0.001), and during control than rest (*p* < 0.001). Furthermore, a significant interaction was seen for “regulation-by-group” (F1; 76 = 7.995, *p* = 0.007, ηp^2^ = 0.174) (Figure 5). For HC, activation was higher in the regulation block than the non-regulation block (*p* < 0.001), whereas for SZP, activation between blocks did not differ (*p* = 0.965). Furthermore, SZP showed higher activation of the right IFG/aI than HC in the non-regulation block (*p* = 0.005), whereas no group difference appeared for the regulation block (*p* = 0.562).

## 4. Discussion

The current study investigated neural and subjective reactions to the regulation of negative, challenging stress in schizophrenia patients and healthy controls. Gaining more insight into stress regulation in schizophrenia has crucial implications for future research. Disentangling aberrant stress processing in this disorder can help develop adequate stress intervention trainings for patients. In the current study, using a cognitively demanding psychosocial stress task, we successfully induced stress in controls and patients, as seen in subjective stress ratings. The current data further indicate that stress reactions increase due to cognitive regulation attempts instead of being reduced by the effort. This fits with previous reports [9,10]. Furthermore, patients diagnosed with schizophrenia show neural hyper-reactions during regulated and non-regulated stress conditions. The details are discussed in the following. 

### 4.1. Group Differences in Non-Regulated Stress

We observed higher subjective ratings (stress and negative affect) before the stress induction in SZP compared to HC. As our participants were familiarized with the paradigm and the application of stress regulation strategies prior to the start of the experiment, higher ratings before stress induction in SZP compared to HC might indicate increased anticipation of stress in patients based on their previous experience and a high stress vulnerability. This result is partly in accordance with previous reports on a higher heart rate before than after stress induction in schizophrenia patients and with higher subjective ratings in the anticipatory phase of a psychosocial stress paradigm in at-risk youths compared to healthy individuals (e.g., [31,32]). Thus, anticipating stressful events serves as a stress induction per se, modulating the stress reaction in schizophrenia patients to a greater extent compared to healthy individuals, possibly due to a higher stress vulnerability and previous experience with stressful events. On a neural level, SZP recruits the left pOP/pI and the right IFG/aI more strongly than HC during non-regulated stress, a pattern that is also visible in negative affect after non-regulated stress. The pOP covers secondary somatosensory regions and is part of a somatosensory processing network [33]. Decreased grey-matter volume of the pOP has been reported in genetically high-risk individuals [34], and the pOP shows reduced functional connectivity with default-mode network nodes in schizophrenia [35]. In our data, patients already showed higher activation in this region during the rest condition as well as specifically during the non-regulated stress condition, potentially indicating heightened processing of somatosensory awareness and negative bodily states during non-regulated stress [29,33]. The right IFG was shown to be relevant for processing negative affective states [29] and emotion regulation in general [36]. Furthermore, in SZP, reduced cortical thickness was reported for the right IFG [37], and it was activated more strongly in a cognitive stress task compared to rest in a previous stress experiment [5]. Decreased activation of the right IFG during down-regulation and increased activation during up-regulation of negative affect were also reported in SZP [11]. Thus, IFG/aI activation is indicating processing, regulating, and suppressing/inhibiting negative, subjective experiences, accompanied by sensoric and affective mapping and evaluation of negative emotions (e.g., [29]). Higher activation of this cluster may indicate increased processing of negative affective states in patients compared to healthy individuals during non-regulated stressful events. 

Thus, in SZP, unregulated stress leads to higher negative affect ratings and higher activation in neural regions associated with processing negative affective and bodily states compared to healthy controls. 

### 4.2. Group Differences in Stress Regulation 

After the regulation block, groups did not show differences in negative affect, and negative affect ratings of SZP did not increase from non-regulated to regulated stress. Contrarily, in HC, negative affect increased with the instruction to regulate negative sensations compared to non-regulated stress, which fits with previous reports in healthy subjects that negative stress reactions might increase due to cognitive regulation attempts instead of being reduced by the effort [9,10]. Notably, no group differences occurred for the self-reported application of general emotion regulation and stress coping traits in everyday life. Thus, although subjective ratings did not decrease in either HC or SZP, in SZP they did not further increase due to the cognitive effort such as regulation attempts, a pattern that was shown for HC. This pattern fits well with the high stress vulnerability that is frequently reported in schizophrenia [2] and might be attributed to an increased reaction due to stress anticipation. 

On a neural level, for pOP/pI a similar pattern appeared as for the subjective ratings, indicating no group difference during stress regulation. Similarly, no group difference emerged for IFG/aI activation during the regulation block. Thus, summarized, SZP did not show a modulation in (i) negative affect and activity of (ii) IFG/aI as well as (iii) pOP/pI due to regulation attempts. They rather showed already higher negative affect and activity of the IFG/aI and pOP/pI than HC in the non-regulation block. In contrast, HC showed increases on these subjective and neural levels from non-regulation to regulation. Our findings provide evidence of a dysfunctional involvement and hyper-activation of stress processing areas in SZP, including the IFG/aI and the pOP/pI, which show higher activation in SZP than in HC in the non-regulation block. Interestingly, there was no further modulation due to regulation attempts in SZP. While HC were significantly more stressed with the instruction to regulate, which was apparent on a subjective and neural level, SZP did not show a modulation due to this instruction and were stressed similarly both times. 

In contrast, SZP showed less deactivation of the ventral ACC than HC in both rest conditions and the control condition of the regulation block. Alterations in ACC activity and connectivity were shown previously in SZP (e.g., [38]). In SZP, activation of the ACC has been reported while maintaining negative affect, and it was positively associated with negative symptom severity [11]. Furthermore, stronger deactivations have been reported in healthy individuals prone to negative symptoms during stress processing compared to controls [6]. Although in our exploratory analyses no significant associations between ACC activity and symptom severity emerged (see Supplementary Materials), future studies might want to further investigate this link between stress processing and symptom dimensions via confirmatory study designs. Furthermore, a previous meta-analysis documented that a reduction of deactivations in the ventral ACC is seen at the beginning of the disease [39], and this region is further associated with genotype-supported psychosis risk variants [40,41]. In our study, less deactivation of the ACC in the regulation block compared to the non-regulation block indicated that patients already failed to down-regulate during the stress-free control condition, potentially due to the diseases specific aberrant function and connectivity in this region. Taken together with the aberrant activation of the IFG/aI and the pOP/pI during stress in SZP, we assume that, on a neural level, stress and the attempt to regulate it increased processing of somatosensory awareness and negative affective states in SZP. 

Interestingly, a meta-analysis on the neural effects of psychotherapy in mental disorders revealed the right IFG and the ACC as two of the core regions being modulated by psychotherapeutic interventions [42], showing less activation after treatment than before. Our study shows that these neural regions are also significantly involved in stress processing and cognitive regulation of stress in schizophrenia patients. These regions might be crucial treatment nodes for neuromodulation studies (see also [43]) for patients vulnerable to stress from a transdiagnostic perspective. 

### 4.3. Limitations and Future Directions 

Our data have some limitations that may influence data interpretation and raise ideas for future research. All patients were medicated, and we cannot exclude the influence of medication on our data, although no significant associations occurred with OLZ-equivalents (see Supplementary Materials). 

Despite performing a stress induction during fMRI with individuals diagnosed with schizophrenia, which poses major challenges, we nevertheless acknowledge the small sample size, which should be taken into consideration when interpreting the data. Determining the power a-posteriori (G*Power [44], *n* = 40, 2 groups, 4 measurements, effect size f: 0.28; alpha error probability: 0.05) revealed an achieved power of more than 99% for the reported significant results.

Sex is a significantly contributing factor to stress reactivity, emotion regulation, and prevalence rates in mental disorders (e.g., [10,45,46,47]). Although the current groups were matched for equal sex distribution, the sample size was too small to additionally include sex as a factor in our analyses.

The current data, taken together with previous reports, indicate that cognitive regulation in challenging, cognitively demanding, and stressful contexts does not reduce stress reactions and, therefore, is not the intervention of choice for effective stress regulation. Longer-lasting trainings and interventions targeting, e.g., self-esteem might modulate feelings of imperturbation within challenging situations and thereby indirectly regulate stress reactions more appropriately. Future studies should assess whether other regulation strategies and neural modulation of specific regions are useful for decreasing stress reactions in cognitively challenging contexts.

## 5. Conclusions

This study provides evidence of altered involvement of brain areas included in stress processing and emotion regulation, such as the IFG/aI, the pOP/pI, and the ACC in SZP. On a subjective level, SZP reported higher stress anticipation and higher stress reactions to non-regulated stress, whereas groups did not differ following regulated stress. This indicates a missing modulation in SZP due to stress regulation. During non-regulated stress, higher neural activation in SZP than in HC was seen in the pOP/pI and the IFG/aI, both regions associated with processing negative affective and bodily states. This group difference was not apparent in the regulation block, though SZP showed less deactivation in the ACC during the stress-free control condition in the regulation block, potentially due to a disease-specific aberrant functioning and connectivity of this region. 

Taken together, our results indicate that in healthy individuals, cognitive regulation of stress increases subjective and neural stress reactions. In SZP, however, unregulated stress already leads to hyperreactions, and regulation attempts did not improve these. Considering the tight link between schizophrenia and stress reactions, the investigation of neural stress regulation stimulates new treatment approaches, including neuromodulation techniques that might efficiently foster stress regulation competencies. The ability to successfully regulate one’s stress reaction might be a necessary prerequisite to disrupt the vicious circle of long-term dependence and re-admission of patients diagnosed with schizophrenia. Further, to address and therapeutically target these abilities may help SZP better cope with stressful situations, potentially improve psychopathology, and open the route to social and occupational integration. Thus, the current study establishes a promising research avenue to strengthen patients wellbeing and stress regulation abilities.

## Figures and Tables

**Table 1:** Sample characteristics.

	SZP *n* = 20		HC *n* = 20		
	Mean	SD	Mean	SD	*p*-Value
Age (years)	36.75	10.33	33.75	10.30	0.363
**Working memory (raw score)**	13.55	4.03	18.11 ^a^	4.11	***0.001***
Verbal intelligence (raw score)	29.32 ^a^	8.49	33.05	3.50	0.088
**Processing speed (TMT-A, s)**	27.21	7.36	19.56	8.89	***0.005***
**Cognit. flexibility (TMT-B, s)**	55.58	33.37	30.70	8.97	***0.004***
**BDI-II**	9.85	5.91	2.85	3.08	***<0.001***
Emotion regulation					
Suppression	4.03 ^b^	1.28	4.24 ^a^	1.34	0.631
Reappraisal	4.42 ^b^	0.86	4.50 ^a^	1.40	0.830
Stress coping					
Task oriented stress coping	3.53 ^a^	0.64	3.60	0.65	0.753
Emotion oriented stress coping	2.54 ^a^	0.65	2.49	0.68	0.821
Distraction	2.62 ^a^	0.63	2.18	0.84	0.071
Social diversion	3.12 ^a^	0.83	3.24	0.77	0.644
Avoidance	2.87 ^a^	0.64	2.71	0.58	0.412
Highest educational degree					0.075
Elementary school	0		0		
Secondary modern school	0		0		
Junior high school	2		3		
Vocational diploma	0		10		
Secondary school	5		0		
Academic studies	6		3		
Training	7		4		
Doctorate	0		0		
Clinical parameters					
Age of onset (years)	27.20	7.86			
Duration of illness (years)	9.53	8.66			
Number of episodes	3.28 ^b^	2.47			
Global symptoms	26.95	7.54			
Positive symptoms	15.50	5.93			
Negative symptoms	11.80	4.63			
Negative dimension	4.34	1.60			
Positive dimension	4.86	1.98			
Affective dimension	5.47	1.67			
Cognitive dimension	4.97	2.20			
Global functioning	51.53	13.97			
CPZ-equivalents	536.30 ^b^	275.77			
OLZ-equivalents	20.26 ^b^	9.00			

Note: Working memory: digit span (raw scores; WIE); verbal intelligence (raw scores; WST); processing speed/cognitive flexibility (s; TMT-A/B); depression scores (BDI-II); emotion regulation: ERQ; stress coping: CISS; symptoms: PANSS; dimensions: [23]; global functioning: global assessment of functioning scale. ^a^ *n* = 19, ^b^ *n* = 18, SD = standard deviation.

**Table 2:** Subjective ratings.

	SZP		HC	
	Pre-Stress	Post-Stress	Pre-Stress	Post-Stress
**Subjective stress**				
Non-regulation	1.68 (0.88)	2.74 (1.24)	1.33 (0.59)	2.78 (1.22)
Regulation	2.05 (1.18)	2.58 (1.26)	1.28 (0.67)	2.89 (1.28)
**Negative affect**				
Non-regulation	13.47 (4.10)	17.79 (7.00)	10.11(3.71)	13.06 (5.90)
Regulation	14.11 (4.65)	17.05 (6.23)	12.00 (3.22)	17.50 (6.72)

Note: The table is indicating mean ratings (standard deviation) for subjective stress and negative affect. SZP: n = 19, HC: n = 18.

## Data Availability

Due to missing consent forms for public data sharing, the data presented in this study are available on request from the corresponding author.

## Supplementary Materials

The following supporting information can be downloaded at: https://www.mdpi.com/article/10.3390/jcm12072749/s1, File S1: Material and methods, Results, Figure S1: Subjective ratings. [48,49,50,51]; Table S1: Whole-brain analysis [33,52,53,54,55,56].

## References

[1] Kirschbaum C., Pirke K.M., Hellhammer D.H. (1993). The ‘Trier Social Stress Test’—A Tool for Investigating Psychobiological Stress Responses in a Laboratory Setting. Neuropsychobiology.

[2] Walker E.F., Diforio D. (1997). Schizophrenia: A Neural Diathesis-Stress Model. J. Psychol. Rev..

[3] Myin-Germeys I., van Os J., Schwartz J.E., Stone A.A., Delespaul P.A. (2001). Emotional Reactivity to Daily Life Stress in Psychosis. Arch. Gen. Psychiatry.

[4] Aas M., Dazzan P., Mondelli V., Toulopoulou T., Reichenberg A., Di Forti M., Fisher H.L., Handley R., Hepgul N., Marques T. (2011). Abnormal Cortisol Awakening Response Predicts Worse Cognitive Function in Patients with First-Episode Psychosis. J. Psychol. Med..

[5] Castro M.N., Villarreal M.F., Bolotinsky N., Papávero E., Goldschmidt M.G., Costanzo E.Y. (2015). Brain Activation Induced by Psychological Stress in Patients with Schizophrenia. Schizophr. Res..

[6] Soliman A., O’Driscoll G.A., Pruessner J., Joober R., Ditto B., Streicker E., Goldberg Y., Caro J., Rekkas P.V., Dagher A. (2011). Limbic Response to Psychosocial Stress in Schizotypy: A Functional Magnetic Resonance Imaging Study. J. Schizophr. Res..

[7] Ochsner K.N., Ray R.D., Cooper J.C., Robertson E.R., Chopra S., Gabrieli J.D.E., Gross J.J. (2004). For Better or for Worse: Neural Systems Supporting the Cognitive down- and up-Regulation of Negative Emotion. Neuroimage.

[8] Berking M., Wupperman P. (2012). Emotion Regulation and Mental Health: Recent Findings, Current Challenges, and Future Directions. Curr. Opin. Psychiatry.

[9] Raio C.M., Orederu T.A., Palazzolo L., Shurick A.A., Phelps E.A. (2013). Cognitive Emotion Regulation Fails the Stress Test. Proc. Natl. Acad. Sci. USA.

[10] Kogler L., Gur R.C., Derntl B. (2015). Sex Differences in Cognitive Regulation of Psychosocial Achievement Stress: Brain and Behavior. Hum. Brain Mapp..

[11] Morris R.W., Sparks A., Mitchell P.B., Weickert C.S., Green M.J. (2012). Lack of Cortico-Limbic Coupling in Bipolar Disorder and Schizophrenia during Emotion Regulation. Transl. Psychiatry.

[12] Perry Y., Henry J.D., Grisham J.R. (2011). The Habitual Use of Emotion Regulation Strategies in Schizophrenia. Br. J. Clin. Psychol..

[13] van der Meer L., Swart M., van der Velde J., Pijnenborg G., Wiersma D., Bruggeman R., Aleman A. (2014). Neural Correlates of Emotion Regulation in Patients with Schizophrenia and Non-Affected Siblings. PLoS ONE.

[14] Wittchen H.-U., Zaudig M., Fydrich T. (1997). Strukturiertes Klinisches Interview Für DSM-IV.

[15] Oldfield R.C. (1971). The Assessment and Analysis of Handedness: The Edinburgh Inventory. Neuropsychologia.

[16] Schmidt K.H., Metzler P. (1992). Wortschatztest (WST).

[17] Reitan R. (1956). Trail Making Test: Manual for Administration, Scoring and Interpretation.

[18] Aster M., Neubauer A., Horn R. (2006). Wechsler Intelligenztest Für Erwachsene (WIE). Deutschsprachige Bearbeitung Und Adaption Des WAIS-III von David Wechsler.

[19] Kälin W., von Endler N.S., Parker J.D.A. (1995). Deutsche 24-Item Kurzform Des “Coping Inventory for Stressful Situations” (CISS).

[20] Abler B., Kessler H. (2009). Emotion Regulation Questionnaire–Eine Deutschsprachige Fassung Des ERQ von Gross Und John. Diagnostica.

[21] Hautzinger M., Keller F., Kühner C. (2006). Beck Depression Inventar II (BDI 2).

[22] Kay S.R., Fiszbein A., Opler L.A. (1987). The Positive and Negative Syndrome Scale (PANSS) for Schizophrenia. Schizophr. Bul..

[23] Chen J., Patil K.R., Weis S., Sim K., Nickl-Jockschat T., Zhou J., Aleman A., Sommer I.E., Liemburg E.J., Hoffstaedter F. (2020). Neurobiological Divergence of the Positive and Negative Schizophrenia Subtypes Identified on a New Factor Structure of Psychopathology Using Non-Negative Factorization: An International Machine Learning Study. Biol. Psychiatry.

[24] Dedovic K., Renwick R., Khalili-Mahani N., Engert V., Lupien S.J., Pruessner J.C. (2005). The Montreal Imaging Stress Task: Using Functional Imaging to Investigate the Effects of Perceiving and Processing Psychosocial Stress in the Human Brain. J. Psychiatry Neurosci..

[25] Dedovic K., Rexroth M., Wolff E., Duchesne A., Scherling C., Beaudry T., Lue S.D., Lord C., Engert V., Pruessner J.C. (2009). Neural Correlates of Processing Stressful Information: An Event-Related FMRI Study. Brain Res..

[26] Watson D., Clark L.A., Tellegen A. (1988). Development and Validation of Brief Measures of Positive and Negative Affect: The PANAS Scales. J. Pers. Soc. Psychol..

[27] Ashburner J., Friston K.J. (2005). Unified Segmentation. Neuroimage.

[28] Eickhoff S.B., Stephan K.E., Mohlberg H., Grefkes C., Fink G.R., Amunts K., Zilles K. (2005). A New SPM Toolbox for Combining Probabilistic Cytoarchitectonic Maps and Functional Imaging Data. Neuroimage.

[29] Kogler L., Müller V.I., Chang A., Eickhoff S.B., Fox P.T., Gur R.C., Derntl B. (2015). Psychosocial versus Physiological Stress-Meta-Analyses on the Deactivations and Activations of the Neural Correlates of Stress Reactions. Neuroimage.

[30] Tischler L., Petermann F., Trail Making Test (TMT) (2010). Zeitschrift für Psychiatrie, Psychologie und Psychotherapie. Z. Psychiatr. Psychol. Psychother..

[31] Rokita K.I., Dauvermann M.R., Mothersill D., Holleran L., Bhatnagar P., McNicholas Á., McKernan D., Morris D.W., Kelly J., Hallahan B. (2021). Current psychosocial stress, childhood trauma and cognition in patients with schizophrenia and healthy participants. Schizophr. Res..

[32] Carol E.E., Spencer R.L., Mittal V.A. (2021). Acute Physiological and Psychological Stress Response in Youth at Clinical High-Risk for Psychosis. Front. Psychiatry.

[33] Eickhoff S.B., Amunts K., Mohlberg H., Zilles K. (2006). The Human Parietal Operculum. II. Stereotaxic Maps and Correlations with Functional Imaging Results. Cereb. Cortex.

[34] Chang M., Womer F.Y., Bai C., Zhou Q., Wei S., Jiang X., Geng H., Zhou Y., Tang Y., Wang F. (2016). Voxel-Based Morphometry in Individuals at Genetic High Risk for Schizophrenia and Patients with Schizophrenia during Their First Episode of Psychosis. PLoS ONE.

[35] Schilbach L., Hoffstaedter F., Müller V.I., Cieslik E.C., Goya-Maldonado R., Trost S., Sorg C., Riedl V., Jardri R., Sommer I. (2015). Transdiagnostic Commonalities and Differences in Resting State Functional Connectivity of the Default Mode Network in Schizophrenia and Major Depression. NeuroImage Clin..

[36] Morawetz C., Bode S., Derntl B., Heekeren H.R. (2017). The Effect of Strategies, Goals and Stimulus Material on the Neural Mechanisms of Emotion Regulation: A Meta-Analysis of FMRI Studies. Neurosci. Biobehav. Rev..

[37] Massey S.H., Stern D., Alden E.C., Petersen J.E., Cobia D.J., Wang L., Csernansky J.G., Smith M.J. (2017). Cortical Thickness of Neural Substrates Supporting Cognitive Empathy in Individuals with Schizophrenia. Schizophr. Res..

[38] Rolls E.T., Cheng W., Gilson M., Gong W., Deco G., Lo C.Z., Yang A.C., Tsai S., Liu M., Lin C. (2019). Beyond the Disconnectivity Hypothesis of Schizophrenia. Cereb Cortex.

[39] Radua J., Borgwardt S., Crescini A., Mataix-Cols D., Meyer-Lindenberg A. (2012). Multimodal Meta-Analysis of Structural and Functional Brain Changes in First Episode Psychosis and the Effects of Antipsychotic Medication. Neurosci. Biobehav. Rev..

[40] Ohi K., Hashimoto R., Yasuda Y., Nemoto K., Ohnishi T. (2012). Impact of the Genome Wide Supported NRGN Gene on Anterior Cingulate Impact of the Genome Wide Supported NRGN Gene on Anterior Cingulate Morphology in Schizophrenia. PLoS ONE.

[41] Rietschel M., Mattheisen M., Degenhardt F., Investigators G., Mu T.W., Czerski P.M., Giegling I., Strengman E., Schmael C., Mors O. (2012). Association between Genetic Variation in a Region on Chromosome 11 and Schizophrenia in Large Samples from Europe. Mol. Psychiatry.

[42] Marwood L., Wise T., Perkins A.M., Cleare A.J. (2018). Meta-Analyses of the Neural Mechanisms and Predictors of Response to Psychotherapy in Depression and Anxiety. Neurosci. Biobehav. Rev..

[43] Wada M., Noda Y., Iwata Y., Tsugawa S., Yoshida K., Tani H., Hirano Y., Koike S., Sasabayashi D., Katayama H. (2022). Dopaminergic dysfunction and excitatory/inhibitory imbalance in treatment-resistant schizophrenia and novel neuromodulatory treatment. Mol. Psychiatry.

[44] Faul F., Erdfelder E., Lang A.-G., Buchner A. (2007). G*Power 3: A flexible statistical power analysis program for the social, behavioral, and biomedical sciences. Behav. Res. Methods.

[45] WHO (2002). Gender in Mental Health Research.

[46] Hodes G.E., Epperson C.N. (2019). Sex Differences in Vulnerability and Resilience to Stress Across the Life Span. Biol. Psychiatry.

[47] Gur R.E., Moore T.M., Rosen A.F.G., Barzilay R., Roalf D.R., Calkins M.E., Ruparel K., Scott J.C., Almasy L., Satterthwaite T.D. (2019). Burden of Environmental Adversity Associated with Psychopathology, Maturation, and Brain Behavior Parameters in Youths. JAMA Psychiatry.

[48] King A., Liberzon I. (2009). Assessing the neuroendocrine stress response in the functional neuroimaging context. Neuroimage.

[49] Boucsein W., Fowles D., Christie M., Grimnes S., Ben-Shakhar G., Roth W.T., Dawson M.E., Filion D.L. (2012). Publication recommendations for electrodermal measurements. Psychophysiology.

[50] Benedek M., Kaernbach C. (2010). A continuous measure of phasic electrodermal activity. J. Neurosci. Methods.

[51] Hemmerich W. (2016). StatistikGuru: Rechner zur Adjustierung des α-Niveaus. https://statistikguru.de/rechner/adjustierung-des-alphaniveaus.html.

[52] Eickhoff S.B., Schleicher A., Zilles K., Amunts K. (2006). The human parietal operculum. I. Cytoarchitectonic mapping of subdivisions. Cereb. Cortex.

[53] Geyer S., Schormann T., Mohlberg H., Zilles K. (2000). Areas 3a, 3b, and 1 of human primary somatosensory cortex. Part 2. Spatial normalization to standard anatomical space. Neuroimage.

[54] Caspers S., Geyer S., Schleicher A., Mohlberg H., Amunts K., Zilles K. (2006). The human inferior parietal cortex: Cytoarchitectonic parcellation and interindividual variability. Neuroimage.

[55] Bludau S., Eickhoff S.B., Mohlberg H., Caspers S., Laird A.R., Fox P.T., Schleicher A., Zilles K., Amunts K. (2014). Cytoarchitecture, probability maps and functions of the human frontal pole. Neuroimage.

[56] Amunts K., Schleicher A., Bürgel U., Mohlberg H., Uylings H.B., Zilles K. (1999). Broca’s region revisited: Cytoarchitecture and intersubject variability. J. Comp. Neurol..

