# Supplementary material for: Cognitive Stress Regulation in Schizophrenia Patients and Healthy Individuals: Brain and Behavior
Source: J Clin Med. 2023 Apr 6;12(7):2749. doi: 10.3390/jcm12072749 (PMC10095473; doi:10.3390/jcm12072749)
Supplement: Supplementary file 1 [file jcm-12-02749-s001.zip › File S1+Figure S1.pdf]

Cognitive stress regulation in schizophrenia patients and healthy individuals: brain and behavior.

Material and methods

Salivary cortisol samples. Saliva samples were taken at six time points using salivettes that participants placed in their mouth. Cortisol was analysed in the clinical trial centre (University Hospital, RWTH Aachen) using "ECLIAs" electrochemiluminessenz immunoassay (functional sensitivity<8.5nml/l). Data were normalized using a log transformation (y=log10(x+1)) prior to statistical analysis [48]. Measurements were controlled for time of last meal, smoking, and circadian rhythm (samples were only taken in the afternoon).

Skin Conductance Response (SCR). Skin conductance data as a measure of peripheral arousal were assessed during neuroimaging. Measurement and analysis of SCR were performed based on previous publication recommendations [49]. Two silver-silver chloride (Ag-AgCl) electrodes were placed at the middle phalanges of the index and middle finger of the left hand. The electrodes were filled with electrode gel (Biopac Systems, Goleta, US). SCR data were recorded at a sampling rate of 5000Hz in DC mode using a bipolar BrainAmp ExG MR amplifier (Brain Products, Gilching, Germany). Data were analysed offline, including down-sampling to 2Hz, artifact reduction using spline interpolation, and extraction of phasic components from tonic activity based on continuous decomposition analysis [50] implemented in Ledalab© software (Leipzig, Germany). Phasic SCR responses were defined as deflections above 0.02µS and were analysed with respect to the parameter "nSCR" (number of SCRs) in a time window of 1–72sec after stimulus onset. Data were normalized using a log transformation (y=log10(x+1)) prior to statistical analysis as done in previous studies [10,49].

Impact of subjective stress, cortisol, and psychopathology on neural activation: regression analyses. To better characterize brain activation, we performed linear regression analyses (hierarchical entry) with the ROI activation (IFG/aI, ACC, hippocampus, amygdala) during stress (non-regulation/regulation) as outcome variable. As predictors group, subjective stress, and cortisol values (each post-non-regulation and post-regulation blocks) were included for the whole sample (SZP and

HC) and medication (OLZ-value) and symptoms (PANSS-values) in an additional analysis only including SZP. Results of these analyses were corrected for multiple comparisons [51].

Results

Physiological responses

Cortisol response. 7 participants (6 SZP) were excluded due to technical problems of data analysis in the clinical trial centre, thus cortisol data of 33 participants (19 HC, 14 SZP) were analysed. A 2x2x2 rmANOVAs with the within-subject factors “regulation” (non-regulation/regulation) and “time” (pre-stress/post-stress), the between-subjects factor “group” and the covariate “processing speed” (TMT-B values), revealed a significant interaction for “regulation-by-group” ($F_{1;30}$=5.941, p=.021, η_p^2=.165), with HC having higher cortisol release in the regulation than the non-regulation block (t_{18}=-2.539, p=.021), whereas no such difference appeared in SZP (t_{13}=.876, p=.397). Groups neither differed in the non-regulation nor the regulation block (both ps>.207). Additionally, the interaction “time-by-group” was significant ($F_{1;30}$=4.318, p=.046, η_p^2=.126), with HC showing a decrease in cortisol from before to after stress (t_{18}=2.300, p=.034), while no such difference appeared in SZP (t_{13}=-.739, p=.473). Groups neither differed before nor after stress (both ps>.234). No other main effect or interaction was significant (all ps>.69).

Skin conductance response (SCR). Individual SCR data was analysed with a 2x2x2 rmANOVA with "regulation" (non-regulation, regulation) and "condition" (control, stress) as within-subject factors, “group” as the between-subjects factor and “processing speed” (TMT-B values) as covariate. Due to severe artefacts and a broken wire, data from 16 participants had to be excluded from SCR analysis, leaving 23 participants for the analysis (15 HC; 8 SZP). nSCR revealed no significant main effects (regulation: $F_{1;20}$=1.987, p=.174, η_p^2=.090; condition: $F_{1;20}$=2.261, p=.117, η_p^2=.102; group: $F_{1;20}$=0.124, p=.728, η_p^2=.006) or interactions (all ps>.12).

Region-of-interest analyses.

Hippocampus. The rmANOVA with the within-subjects factors „laterality“ (left/right), „regulation“ (non-regulation/regulation), „condition“ (rest/control/stress) and the between-subjects factor „group“ revealed significant main effects for „laterality“ ($F_{1;76}$=11.493, *p*=.002, ηp^2=0.232) with less deactivation in the right than the left hippocampus, „regulation“ ($F_{1;76}$=5.034, *p*=.031, ηp^2=0.117) with higher activation in the non-regulation than the regulation block and for “group” ($F_{1;76}$=6.746, *p*=.013, ηp^2=0.151) with higher activation in SZP than HC. Furthermore, significant interactions were found for „laterality-by-condition“ ($F_{1;76}$=11.579, *p*<.001, ηp^2=0.234) and „regulation-by-condition“ ($F_{1;76}$=7.433, *p*=.001, ηp^2=0.164). Exploring the “regulation-by-condition” interaction, post-hoc t-tests revealed higher hippocampus activation for the rest condition in the non-regulation compared to the regulation block (t_{39}=3.998, *p*<.001). Control and stress condition did not differ between the non-regulation and the regulation block (both ps>.288). Furthermore, within the regulation block, the hippocampus was more strongly activated in the stress (t_{39}=3.709, *p*=.001) and less deactivated in the control (t_{39}=3.545, *p*=.001) compared to the rest condition. Control and stress condition within the regulation block did not differ from each other (t_{39}=0.933, *p*=.356) and all conditions within the non-regulation block did not differ from each other (all *p*s>.240). Post-hoc t-tests exploring the “laterality-by-condition” interaction showed higher activation in the right than the left hippocampus for the control (t_{39}=3.474, *p*=.001) and the stress condition (t_{39}=4.447, *p*<.001), but no differences in laterality for the rest condition (t_{39}=1.022, *p*=.313). No differences appeared within left or right hippocampus between the three conditions (all *p*s>.064).

Amygdala. The rmANOVA revealed a significant main effect for „laterality“ ($F_{1;76}$=6.417, *p*=.016, ηp^2=0.144) with less deactivation in the right than the left amygdala and „condition“ ($F_{1;76}$=8.221, *p*=.001, ηp^2=0.178) with higher deactivation in the control compared to the stress (*p*=.011) and the rest (*p*<.001) condition. Furthermore, a significant „laterality-by-group“ interaction emerged ($F_{1;76}$=4.446, *p*=.042, ηp^2=0.105). Post-hoc paired t-tests indicated that in HC, less deactivation was seen in the right than the left amygdala (t_{19}=3.059, *p*=.006), whereas in SZP deactivation of the left and right amygdala did not differ (t_{19}=0.049, *p*=.965). Furthermore, SZP showed less deactivation than HC in the left

amygdala (t_{38}=-2.187, p=.035) but no group difference appeared for the right amygdala (t_{38}=-0.847, p=.403).

Regression analyses. Regression analyses did not show any significant results after correction for multiple comparison.

Figure S1. Subjective ratings.

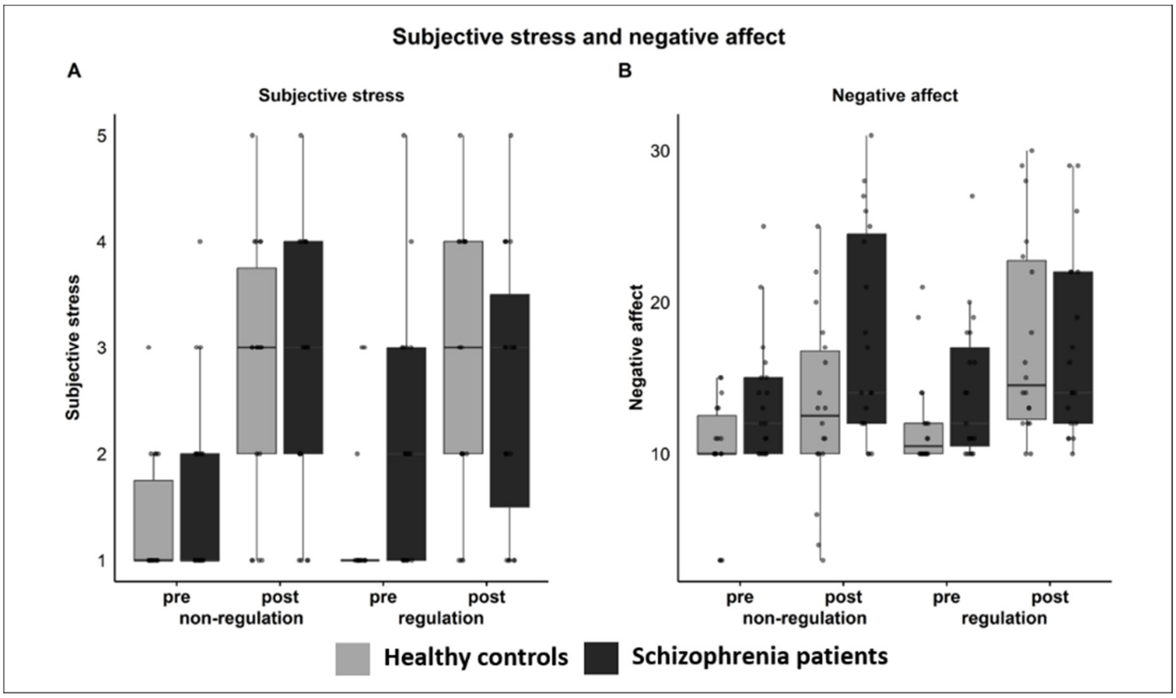

Figure S1. *Subjective ratings. (A) Subjective stress, interaction "time-by-group": SZP compared to HC reported higher subjective stress before the stress paradigm. Subjective stress increased in both groups from before to after the stress paradigm. (B) Negative affect, interaction "regulation-by-time-by-group": pre-stress, a group main effect appeared with SZP reporting higher negative affect before the stress paradigm than HC. Post-stress, an interaction "regulation-by-group" appeared with SZP indicating higher negative affect than HC after the non-regulation block. No difference appeared after the regulation block. HC reported higher negative affect after the regulation compared to the non-regulation block. non-reg=non-regulation block; reg=regulation block.*
