# Supplementary material for: Cognitive Stress Regulation in Schizophrenia Patients and Healthy Individuals: Brain and Behavior
Source: J Clin Med. 2023 Apr 6;12(7):2749. doi: 10.3390/jcm12072749 (PMC10095473; doi:10.3390/jcm12072749)
Supplement: Supplementary file 1 [file jcm-12-02749-s001.zip › Table S1.pdf]

**Cognitive stress regulation in schizophrenia patients and healthy individuals: brain and behavior.**

**Supplementary material Table S1. Whole brain analysis.**

**Table S1.**

| Contrast | Cluster size | Macronanatomical location | Cytoarchitectonical activation | X | Y | Z | t value |
|---|---|---|---|---|---|---|---|
| **Healthy controls Non-regulation Control > stress** | | | | | | | |
| | 112 | | | | | | |
| | | R Precuneus | | 6 | -48 | 22 | STAT = 3.99 |
| **Healthy controls Non-regulation Stress > control** | | | | | | | |
| | 119 | | | | | | |
| | | N/A | | 14 | 26 | -4 | STAT = 4.87 |
| | | R Olfactory cortex | Area 33 | 4 | 20 | 0 | STAT = 4.43 |
| **Healthy controls Regulation Control > stress** | | | | | | | |
| | | non significant | | | | | |
| **Healthy controls Regulation Stress > control** | | | | | | | |
| | 180 | | | | | | |
| | | L Middle Frontal Gyrus | | -28 | -12 | 56 | STAT = 4.03 |
| | | L Precentral Gyrus | | -34 | -8 | 60 | STAT = 3.78 |
| | | N/A | | -20 | -14 | 58 | STAT = 3.66 |
| | | N/A | | -14 | -12 | 58 | STAT = 3.65 |
| | | L Precentral Gyrus | | -36 | -14 | 60 | STAT = 3.53 |

| | | | | | | |
|---|---|---|---|---|---|---|
| **Healthy controls Non-regulation > regulation** | | non significant | | | | |
| **Healthy controls Regulation > non-regulation** | | | | | | |
| | 1622 | | | | | |
| | | L Paracentral Lobule | -10 | -32 | 56 | STAT = 6.08 |
| | | R Posterior-Medial Frontal | 12 | -30 | 54 | STAT = 5.60 |
| | | R Paracentral Lobule Area 4a | 8 | -32 | 68 | STAT = 4.94 |
| | | L MCC | -12 | 0 | 46 | STAT = 4.91 |
| | | L Superior Medial Gyrus | -10 | 24 | 46 | STAT = 4.84 |
| | | N/A | -12 | 20 | 44 | STAT = 4.74 |
| | | N/A | -14 | 8 | 48 | STAT = 4.34 |
| | | L MCC | -12 | -22 | 48 | STAT = 4.24 |
| | | L MCC | -10 | -14 | 56 | STAT = 4.19 |
| | | L MCC | 12 | -16 | 50 | STAT = 4.10 |
| | | L MCC | 6 | -22 | 48 | STAT = 3.66 |
| | 621 | | | | | |
| | | N/A | 26 | 6 | 30 | STAT = 4.55 |
| | | R Rolandic Operculum | 46 | -18 | 26 | STAT = 4.46 |
| | | N/A | 32 | -36 | 10 | STAT = 4.42 |
| | | N/A | 24 | -12 | 28 | STAT = 4.35 |
| | | N/A | 34 | -14 | 26 | STAT = 4.21 |
| | | N/A | 22 | -8 | 24 | STAT = 4.18 |
| | | R IFG (p. Triangularis) | 46 | 16 | 30 | STAT = 4.10 |
| | | R Caudate Nucleus | 22 | 14 | 18 | STAT = 4.08 |
| | | R IFG (p. Triangularis) | 32 | 16 | 32 | STAT = 3.94 |
| | | R IFG (p. Triangularis) | 52 | 20 | 32 | STAT = 3.93 |

| | | | | | | | | |
|---|---|---|---|---|---|---|---|---|
| | | N/A | | 22 | -18 | 28 | STAT = 3.90 |
| | 554 | | | | | | |
| | | N/A | | -20 | 8 | 20 | STAT = 4.91 |
| | | N/A | | -20 | -22 | 26 | STAT = 4.55 |
| | | N/A | Thal: Parietal | -28 | -32 | 8 | STAT = 4.38 |
| | | N/A | | -20 | -2 | 24 | STAT = 4.34 |
| | | N/A | | -28 | -42 | 18 | STAT = 4.33 |
| | | N/A | | -30 | -42 | 14 | STAT = 4.20 |
| | | N/A | Thal: Parietal | -24 | -30 | 18 | STAT = 3.80 |
| | | N/A | | -22 | 16 | 28 | STAT = 3.77 |
| | | L Hippocampus | | -30 | -26 | -4 | STAT = 3.50 |
| | | N/A | | -28 | -4 | 26 | STAT = 3.45 |
| | | L Hippocampus | | -32 | -32 | 0 | STAT = 3.32 |
| | 296 | | | | | | |
| | | L IFG (p. Orbitalis) | | -30 | 30 | -2 | STAT = 5.28 |
| | | L IFG (p. Orbitalis) | | -22 | 30 | -2 | STAT = 4.90 |
| | | L Caudate Nucleus | | -10 | 20 | 4 | STAT = 4.11 |
| | | N/A | | -18 | 40 | -4 | STAT = 3.49 |
| | 241 | | | | | | |
| | | L Middle Frontal Gyrus | | -34 | 40 | 24 | STAT = 5.11 |
| | | N/A | | -28 | 32 | 20 | STAT = 4.17 |
| **Schizophrenia patients Non-regulation Control > stress** | | non significant | | | | | |

| | | | | | |
|---|---|---|---|---|---|
| **Schizophrenia patients Non-regulation Stress > control** | non significant | | | | |
| **S Schizophrenia patients Regulation Control > stress** | non significant | | | | |
| **Schizophrenia patients Regulation Stress > control** 134 | L Precentral Gyrus | -28 | -12 | 54 | STAT = 3.78 |
| | L Precentral Gyrus | -34 | -14 | 52 | STAT = 3.72 |
| | L Precentral Gyrus | -36 | -14 | 60 | STAT = 3.49 |
| | L Precentral Gyrus | -32 | -8 | 60 | STAT = 3.26 |
| | N/A | -24 | -16 | 44 | STAT = 3.19 |
| **Schizophrenia patients Non-regulation > regulation** | non significant | | | | |
| **Schizophrenia patients Regulation > non-regulation** 281 | L Mid Orbital Gyrus | 0 | 40 | -8 | STAT = 4.38 |
| | R Mid Orbital Gyrus | 10 | 42 | -8 | STAT = 4.10 |
| | N/A | 18 | 32 | 4 | STAT = 3.77 |

| | | | | | | |
|---|---|---|---|---|---|---|
| | N/A | | 20 | 38 | -2 | STAT = 3.67 |
| | N/A | | 16 | 42 | -2 | STAT = 3.66 |
| | R Rectal Gyrus | Area Fo1 | 6 | 46 | -14 | STAT = 3.27 |
| 167 | | | | | | |
| | R Middle Frontal Gyrus | | 44 | 22 | 44 | STAT = 4.37 |
| | R Middle Frontal Gyrus | | 36 | 20 | 50 | STAT = 4.22 |
| | R Middle Frontal Gyrus | | 34 | 30 | 46 | STAT = 4.01 |
| | R Middle Frontal Gyrus | | 44 | 16 | 46 | STAT = 3.99 |
| | R Middle Frontal Gyrus | | 30 | 16 | 54 | STAT = 3.81 |
| | R Middle Frontal Gyrus | | 42 | 28 | 42 | STAT = 3.79 |
| | R Superior Frontal Gyrus | | 26 | 24 | 54 | STAT = 3.48 |
| | R Middle Frontal Gyrus | | 34 | 34 | 44 | STAT = 3.37 |
| 157 | | | | | | |
| | L MCC | | 0 | -46 | 40 | STAT = 4.13 |
| | L Precuneus | | -4 | -66 | 36 | STAT = 4.02 |
| | L PCC | | 0 | -58 | 38 | STAT = 3.69 |
| 149 | | | | | | |
| | L Insula Lobe | | -34 | -28 | 28 | STAT = 3.96 |
| | N/A | | -28 | -22 | 26 | STAT = 3.71 |
| | N/A | | -30 | -36 | 28 | STAT = 3.55 |
| | N/A | | -38 | -22 | 30 | STAT = 3.47 |
| | L Rolandic Operculum | | -40 | -20 | 28 | STAT = 3.26 |
| **Whole-brain F-test Group-by-regulation-by-condition** 147 | | | | | | |

| | | | | | | |
|---|---|---|---|---|---|---|
| | L Rolandic Operculum | Area OP1 [SII] | -46 | -26 | 18 | STAT = 8.19 |
| | L Insula Lobe | Area OP3 [VS] | -34 | -20 | 20 | STAT = 8.14 |
| | L Postcentral Gyrus | Area OP1 [SII] | -52 | -24 | 20 | STAT = 7.49 |

***Supplement Table S1.*** *Brain regions and activation peaks showing whole-brain analyses convergence in activation for healthy controls and schizophrenia patients (control, stress, non-regulation block, regulation block) separately.*
*Note. Coordinates x, y, z of local maxima refer to Montreal Neurological Institute space (MNI). k = number of voxels in cluster. An extent threshold of k=99 contiguous voxels corresponds to a corrected threshold of p<.05.*
*References for histological assignments: OP1, OP 3, OP 4: [33,52]; Area 1: [53]; Area PFt (IPL), Area PGa (IPL), Area PFm (IPL), Area PGp (IPL): [54]; Area Fp2 [55]; Area 45: [56].*
